# Deep Liquid State Machines With Neural Plasticity for Video Activity Recognition

**DOI:** 10.3389/fnins.2019.00686

**Published:** 2019-07-04

**Authors:** Nicholas Soures, Dhireesha Kudithipudi

**Affiliations:** Neuromorphic AI Laboratory, Rochester Institute of Technology, Rochester, NY, United States

**Keywords:** spiking, LSM, local learning, deep, recurrent

## Abstract

Real-world applications such as first-person video activity recognition require intelligent edge devices. However, size, weight, and power constraints of the embedded platforms cannot support resource intensive state-of-the-art algorithms. Machine learning lite algorithms, such as reservoir computing, with shallow 3-layer networks are computationally frugal as only the output layer is trained. By reducing network depth and plasticity, reservoir computing minimizes computational power and complexity, making the algorithms optimal for edge devices. However, as a trade-off for their frugal nature, reservoir computing sacrifices computational power compared to state-of-the-art methods. A good compromise between reservoir computing and fully supervised networks are the proposed deep-LSM networks. The deep-LSM is a deep spiking neural network which captures dynamic information over multiple time-scales with a combination of randomly connected layers and unsupervised layers. The deep-LSM processes the captured dynamic information through an attention modulated readout layer to perform classification. We demonstrate that the deep-LSM achieves an average of 84.78% accuracy on the DogCentric video activity recognition task, beating state-of-the-art. The deep-LSM also shows up to 91.13% memory savings and up to 91.55% reduction in synaptic operations when compared to similar recurrent neural network models. Based on these results we claim that the deep-LSM is capable of overcoming limitations of traditional reservoir computing, while maintaining the low computational cost associated with reservoir computing.

## 1. Introduction

Enabling intelligence on the edge minimizes the round trip delay in decision-making, lowers communication costs, load-balances for the end user, and enhances security with caching or local algorithms to pre-process the data. An emerging input source for edge devices is streaming visual data from first person cameras, such as in smart vehicles, or wearable devices. Being able to accurately process streaming video is crucial for edge devices to understand and react to their environment in a wide range of applications (*eg: path planning, action selection, or surveillance*). A popular application for demonstrating understanding of first-person video data in machine learning and computer vision is video activity recognition. However, majority of state-of-the-art methods for video activity recognition do not target low-end embedded platforms. Complex networks are not amenable for on-device intelligence due to their compute and memory intensive operations (networks with 10–60 million synapses require 0.32–2 GB to store synaptic weights Alom et al., 2018) and long training times (in the order of hours to days with GPUs Fu and Carter, 2016).

In the early 2000s, a computationally light algorithm known as reservoir computing (RC) was proposed by two research groups independently. The two algorithms are otherwise known as the Echo State Network (ESN) (Jaeger, 2001) and the Liquid State Machine (LSM) (Maass et al., 2002). The main difference between the two is that the LSM is a biologically inspired spiking neural network (SNN), whereas the ESN is a rate-based approximation. In this work we focus on the LSM, a neurally inspired algorithm, with innate characteristics for edge devices that bring in size, weight, and power constraints. In particular SNNs can store the neuronal activation's in a single bit (all or nothing signal), can consume as low as ≈20*pJ* per spike (Neftci et al., 2017), and shown to be computationally at least as powerful as sigmoid and threshold neurons (Maass, 1997).

The LSM is a three-layer neural network which consists of an input layer, a liquid layer, and a readout layer. The recurrent connections in the liquid layer allow it to capture dynamic information, where information fades out over time. The advantage of the LSM is that all the synaptic connections, except for those which connect to the readout layer, are randomly initialized and remain fixed. Unique inputs will produce distinct perturbations in the state of the high-dimensional liquid layer from which information can be extracted. By using fixed connections, the LSM can circumvent the need for expensive learning rules and the problem of vanishing gradients which can impede learning with gradient descent approaches in recurrent neural networks. In Soures et al. (2017), it was shown that these networks are robust to internal noise, making them a natural choice for embedded systems, particularly analog implementations which are prone to device noise. However, the conventional LSM model has shown limited applicability in complex real-world problems owing to the single dynamical layer driven by an input signal (Hermans and Schrauwen, 2013; Ma et al., 2017). The single layer constricts the temporal dynamics of the LSM resulting in very large reservoir networks to solve trivial tasks. Another drawback with LSM is its dependence on the initialization of random synaptic connections. Recent literature highlights the gaps in conventional LSM, RC networks in general, and the need to extend the capabilities of these networks (Jaeger, 2007; Triefenbach et al., 2010, 2013; Gallicchio and Micheli, 2016; Wang and Li, 2016; Ma et al., 2017; Bellec et al., 2018). Motivated by these observations, we propose a novel framework that drastically reduces the overall computational resources without sacrificing the overall performance in complex spatiotemporal task. Specific contributions of this work are

1. Deep-LSM, a semi-trained deep spiking recurrent neural network with LSM as a core building block, capable of capturing information over multiple time-scales.
2. Demonstrate that a modular/deep architecture significantly reduces the memory requirements for storing synaptic weights.
3. Use local, unsupervised plasticity mechanisms to partially train the network yields state-of-the-art performance while minimizing the cost of training.
4. Design an attention modulated readout layer to selectively process information in the deep-LSM with limited computational resources.
5. Analyze the model performance on first-person video activity recognition with DogCentric dataset (Iwashita et al., 2014) and demonstrate state-of-the-art performance.
6. Observe ≈ 90% memory savings and reduction in number of operations compared to a LSTM and ≈ 25% reduction of memory consumption in comparison to a standard LSM and 16% decrease in number of operations.

## 2. Related Work

### 2.1. Video Activity Recognition

Egocentric video activity recognition is quickly becoming a pertinent application area due to first person wearable devices such as body cameras or in robotics. In these application domains, real-time learning is critical for deployment beyond controlled environments (such as deep space exploration), or to learn continuously in novel scenarios. Many research groups have focused on solving video activity recognition problems with 2D and 3D convolutions (Tran et al., 2015), optical flow (Simonyan and Zisserman, 2014; Zhan et al., 2014; Ma et al., 2016; Song et al., 2016a), hand-crafted features (Ryoo et al., 2015), combining motion sensors with visual information (Song et al., 2016a,b), or using long-short term memory (LSTM) networks to capture dynamics about spatial information extracted by a convolutional neural network (CNN) (Baccouche et al., 2011; Yue-Hei Ng et al., 2015). These approaches, while befitting for high-end compute platforms, are often not suitable for wearable devices due to the resource intensive networks or the long training times.

Efficient video activity recognition designed for mobile devices has been studied by several research groups. An energy aware training algorithm was proposed in Possas et al. (2018), to demonstrate energy efficient video activity recognition on complex problems. In this work, the authors use reinforcement learning to train a network on both video and motion information captured by sensors while penalizing actions that have high energy costs. Another approach to minimizing energy consumption in mobile devices when using an accelerometer for activity recognition is to minimize the sampling rate (Zheng et al., 2017). In Yan et al. (2012) and Lee and Kim (2016), the authors investigate a network with adaptive features, sampling frequency, and window size for minimizing energy consumption during activity recognition.

Recently Graham et al. (2017) proposed convolutional drift networks (CDNs) for enabling real-time learning on mobile devices. CDNs are an architecture for video activity recognition which use a pre-trained CNN to extract features from video frames and an ESN to capture temporal information. The motivation behind the CDNs is to minimize the training time and compute resources for spatiotemporal tasks when compared to networks akin to LSTMs (Yue-Hei Ng et al., 2015; Graham et al., 2017). A similar sized RC network requires one fourth of the weights, has faster training, and lower energy consumption as that of an LSTM.

### 2.2. Hierarchical Reservoir Computing

As conventional reservoir networks are shallow and capture information in short time-scales, recently several research groups have investigated hierarchical reservoir models. A hierarchical ESN is introduced in Jaeger (2007) with the goal of developing a hierarchical information processing system which feeds on high-dimension time series data and learns its own features and concepts with minimal supervision. The hierarchical layers help the system to process information on multiple timescales where faster information is processed in the earlier layers and information on slower timescales is processed in the final layers. The outputs of each reservoir feed sequentially into the next reservoir in the network. The networks prediction is made from a combination of all the reservoir outputs. More recently, a hierarchical ESN was proposed in Ma et al. (2017). In this work the authors explore the use of trained auto-encoders, principal component analysis, and random connections as encoding layers between each reservoir layer. The downside to this approach is that the output layer is trained on the activity of every encoding layer, the last reservoir, and the current input. This means as the number of layers increases, the output layer size will increase. Another hierarchical model was developed in Triefenbach et al. (2010). This model is implemented by stacking trained ESNs on top of each other to create a hierarchical chain of reservoirs. The hierarchical ESN is applied to speech recognition where the intermediary layers have a readout layer trained to perform the tasks and the inputs to the hierarchical layers are the predictions of the previous layers. With this approach each layer corrects the error from the previous layer. The authors later designed a hierarchical ESN where each layer was trained on a broad representation of the output, which became more specific at later layers (Triefenbach et al., 2013). Another hierarchical ESN proposed in Gallicchio and Micheli (2016) connects an ensemble of ESNs together. In Carmichael et al. (2018), our group has proposed a mod-deepESN architecture, a modular architecture that allows for varying topologies of deep ESNs. Intrinsic plasticity mechanism is embedded in the ESN that contributes more equally toward predictions and achieves better performance with increased breadth and depth. In Wang and Li (2016), a deep LSM model is proposed for image processing which uses multiple LSMs as filters with a single response. The authors use convolution and pooling similar to the process of CNNs and train the LSMs with an unsupervised learning rule. In Bellec et al. (2018), the authors introduce an approximation of backpropagation-through-time for LSMs to optimize the temporal memory of the LSM. The network shows a large improvement in performance on sequential MNIST and speech recognition with the TIMIT speech corpus. Another approach to optimizing the LSM is Roy and Basu (2016), which proposes a computationally efficient on-line learning rule for unsupervised optimization of reservoir connections.

This work aims to develop an algorithm that overcomes few of the gaps in the vanilla RC network while focusing on maintaining the inherent efficiency of LSMs.

## 3. Deep-LSM Model

**Figure 1:**
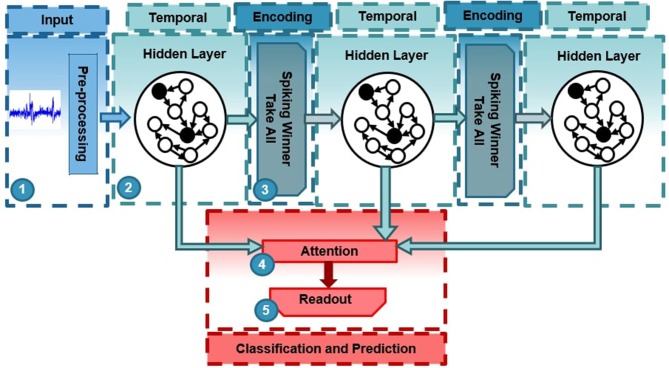
Architecture of deep-LSM with three layers. (1) The input signals are randomly projected to a high dimensional space in the first hidden layer. (2) Hidden layers with random recurrent connections capture temporal information. (3) Spiking winner-take-all layers extract temporal features from the random hidden layers through the deep-LSM. (4) An attention function condensed the representation of the deep-LSM's hidden layers for efficient classification. (5) A readout layer is trained to perform a specific task.

The proposed deep-LSM, shown in Figure 1, is a network comprised of deep randomly initialized hidden layers to capture the key dynamics of input streams. Sandwiched between the hidden layers, unsupervised winner-take-all (WTA) layers encode a low-dimensional representation of the dynamic information captured by the high-dimensional hidden layer. The encoded representation is then passed to the next hidden layer in the network. The main role of the WTA layer is to extract features from the hidden layer to represent its dynamic behavior as a low dimensional input. As data flows through the deep-LSM, different hidden layers process information over multiple time-scales. The main elements of the proposed deep-LSM are optimization of short-term plasticity and initialization of the random hidden layers, the use of spike-timing dependent plasticity (STDP) to implement the unsupervised WTA layers, and the attention modulated readout layer.

### 3.1. Hidden Layer Optimization

The hidden layers in the deep-LSM are similar to the liquid layer in the LSM. The connections between neurons in the input layer to the hidden layer are random and sparse. The probability of a connection is drawn from a uniform random distribution and the degree of sparsity varies based on the application and number of input signals. In Litwin-Kumar et al. (2017), the authors state that the granule cells produce a 10–30x increase in dimensionality. They also highlight that the granule cells need to connect to a sparse number of inputs to produce a unique high-dimensional representation. Using these claims as guiding principles for the initialization of the hidden layer, the number of neurons is set to be approximately 10x the size of the input space in this work. The hidden layer consists of two populations of neurons, primary neurons which are connected to the input layer, and auxiliary neurons which only have recurrent connections within the hidden layer but do not connect to the input layer. Each primary neuron only connects to a sparse number of input neurons, creating a selective response such that no neuron responds to the same feature or set of features. The auxiliary neurons then help to capture dynamic information through their recurrent connections and propagate information through the network.

The hidden layer in this work is implemented with excitatory (E) and inhibitory (I) leaky integrate-and-fire neurons whose dynamics are modeled by (1).

(1) $$\begin{array}{l} \tau \frac{\partial V}{\partial t}=-V+I_{ext}*R, \end{array}$$

When a neuron recieves a pre-synaptic spike, the current is modeled by a square pulse of current with a magnitude proportional to the synaptic strength for 3 ms after the spike occurs. The LIF neurons are instantiated as a 3D grid of neurons with a ratio of 4:1 for the number of excitatory to inhibitory neurons. The probability of a recurrent connection forming is computed by (2).

(2) $$\begin{array}{l} \text{Pr}\left(w_{i,j}^{res}\neq 0\right)=C\text{exp}{\left(-D\left(i,j\right)/\lambda \right)}^{2}, \end{array}$$

Where the probability of a connection depends on a scalar C (determined by the neuron types and the direction of the connection) which sets the maximum probability of a connection, and the Euclidean distance between the neurons scaled by λ which controls how quickly the probability of a connection drops off as the distance increases. The recurrent connections are initialized using fixed weights for each connection type where excitatory to excitatory (EE) connections have a synaptic strength of 3, EI have a strength of 3, IE have a strength of 4, and II have a strength of 1. In Renart et al. (2003) it was shown that neurons having homogeneous excitability is important in the dynamics of temporal memory. To maintain a homogeneous excitability in the hidden layer, the excitatory and inhibitory pre-synaptic connections are normalized so the sum of excitatory synapses and sum of inhibitory synapses is consistent for all neurons.

Another biologically inspired mechanism in the hidden layer is the use of short-term plasticity (STP). STP acts as a form of hidden memory in the hidden layer by reflecting a neurons recent firing activity. It also helps to regulate the overall firing activity by reducing the strength of spikes from highly active neurons. To optimize the STP function for neuromorphic systems, we reduce the computational cost of the STP equations from Markram et al. (1998) to (3) which simplified the model from an exponential function to a simple linear model.

(3) $$\begin{array}{l} S\left(n\right)=S\left(n-1\right)-\alpha *\left(x\left(n\right)-\beta \right) \end{array}$$

where S is the synaptic efficacy regulating the strength of a neurons action potential and is bounded between 0 and 1. If a neuron emits a spike (*x*(*n*) = 1), the strength of S is decreased and if *x*(*n*) = 0 then S is increased. α and β are hyper-parameters used to control the dynamics of STP. A timestep of 1ms is used for all results presented in this work. The benefits of the STP rule in 3 are (i) changes in synaptic efficacy are constant and, (ii) are not dependent on the previous state of the synaptic efficacy.

The outputs of the hidden layer need to be sent to a readout layer to perform classification or prediction. If a binary state matrix (i.e., if a neuron fired) is used to represent the hidden layer's activity, several states collapse upon each other which can impact the networks ability to distinguish the different temporal patterns. Typically an exponential filtering operation is performed on the output of each neuron in the hidden layer (Schrauwen et al., 2007). In this work a synaptic trace operation is implemented at the output of each hidden neuron before transmitting to the readout layer which does not require the computation of any exponential terms. This operation is given by Equation (4)

(4) $$\begin{array}{l} \frac{dX^{trace}}{dn}=\frac{-X^{trace}}{\tau_{trace}}+\underset{n^{f}}{\sum }\delta \left(n-n^{f}\right) \end{array}$$

where the synaptic trace (*X*_*trace*_) keeps track of the behavior of the spike activity of a neuron (x(n)) by increasing the trace by a count of one every time a spike occurs and slowly decaying over time. This trace value is used by the readout layer to perform classification and prediction by capturing the short term behavior of each hidden neuron.

### 3.2. Deep-LSM Implementation

In Jaeger (2007), the authors provide evidence that deep networks are computationally more efficient and powerful than a shallow (single-layer) architecture. A deep model allows the network to learn more complex abstractions of the input and process the input on different timescales in the case of RNNs (Jaeger, 2007). Therefore the deep-LSM can extract higher level temporal features in each subsequent hidden layer before finally sending the information to a readout layer.

The inputs to each layer in the deep-LSM can be described by Equations (5)–(7)

(5) $$\begin{array}{l} I_{L_{1}}\left(n\right)=W_{L_{1}}^{in}*u\left(n\right)+W_{L_{1}}^{rec}*x_{L_{1}}\left(n-1\right) \end{array}$$

(6) $$\begin{array}{l} I_{E_{k}}\left(n\right)=W_{E_{k}}^{in}*x_{L_{l=k}}\left(n\right) \end{array}$$

(7) $$\begin{array}{l} I_{L_{l}}\left(n\right)=W_{L_{l}}^{in}*x_{E_{k=l-1}}\left(n\right)+W_{L_{l}}^{rec}*x_{L_{l}}\left(n-1\right) \end{array}$$

where (5) is the input to the first hidden layer *L*_1_ which combines information from the input layer *u*(*n*) and input from the spiking activity of the hidden layer *x*_*L*_1__ through the recurrent connections. The input to the *k*^*th*^ WTA layer is described by (6) where *x*_*L*_*l* = *k*__(*n*) is the spiking activity of the previous hidden layer. Lastly, (7) is the input to the *l*^*th*^ hidden layer which receives the spiking activity at the current timestep from the previous encoding layer *x*_*E*_*k* = *l*−1__(*n*) and input about the hidden layer's previous spiking activity *x*_*L*_*l*__(*n* − 1) through recurrent connections. In this architecture there is always one more hidden layer than the number of WTA layers because the activity of the hidden layer is what is used for classification.

**Figure 2:**
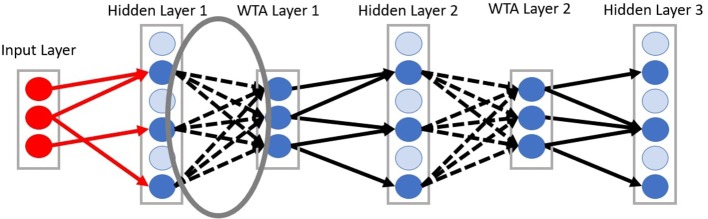
Diagram of connectivity between layers in the deep-LSM. Dashed lines represent connections trained with STDP from a hidden layer to a WTA layer.

In the deep-LSM architecture shown in Figure 2, the synaptic connections from the input layer to the first hidden layer, and from the WTA layers to the hidden layers are sparse. The synaptic connections from the hidden layers to the WTA layers (represented by dashed lines) are fully connected and trained with Spike-time Dependent Plasticity (STDP). STDP is a form of hebbian learning which postulates that neurons which fire together grow together (Hebb, 1949). In this case if a pre-synaptic potential occurs before a post-synaptic potential the synaptic strength is increased and vice-versa, if a post-synaptic potential occurs before a pre-synaptic potential the synaptic strength is decreased.

A simple learning rule based on a pre-synaptic trace from Diehl and Cook (2015) is used to model STDP. The pre-synaptic trace is a function which tracks the recent activity of the pre-synaptic neurons given by (4). The unsupervised learning rule can then be defined as

(8) $$\begin{array}{l} \Delta W_{i,j}=\alpha *\left(X_{j}^{trace}-X^{tar}\right) \end{array}$$

where α is a hyper-parameter to control the magnitude of the weight change. The change in the synaptic strength between pre-synaptic neuron j and post-synaptic neuron i is increased proportional to the difference between the trace of pre-synaptic activity $X_{j}^{trace}$ and the threshold activity level *X*^*tar*^ which determines whether potentiation or depression occurs.

STDP alone can exhibit runaway dynamics which result in synaptic strengths saturating. In order to stabilize the performance of STDP, it is necessary to use the same synaptic scaling function used in the initialization step and intrinsic plasticity (Watt and Desai, 2010). Synaptic scaling normalizes the sum of pre-synaptic connections to α, as shown in (9).

(9) $$\begin{array}{l} W_{i,j}=\frac{W_{i,j}}{\overset{N}{\underset{j=1}{\sum }}W_{i,j}}*\alpha \end{array}$$

Here, the synaptic connection from pre-synaptic neuron j to post-synaptic neuron i (*W*_*i, j*_) is scaled so the total sum of the synaptic connections to neuron i remains constant. This helps stabilize the weights while maintaining the hebbian relation between synapses and removes the effect of noise on the network.

Global inhibition forces unsupervised learning through STDP to generate competition between neurons and causes neurons to learn different patterns. Global inhibition results in a winner-take-all network so that when a neuron fires to a specific pattern, it inhibits all other neurons from firing and learning that same pattern. To prevent a single neuron from constantly inhibiting other neurons, intrinsic plasticity (Watt and Desai, 2010) regulates how often a neuron fires by regulating the neurons firing threshold according to (10)

(10) $$\begin{array}{l} V_{th}=V_{th}+\Theta \end{array}$$

where the neurons firing threshold *V*_*th*_ is increased by Θ and Θ is increased every time a neuron fires and decays back toward its resting value when a neuron does not fire according to a time constant τ shown in (11) (Zhang and Linden, 2003). The increased firing threshold decreases the probability of a neuron spiking multiple times in succession to allow other neurons to learn.

(11) $$\begin{array}{l} \tau \frac{d\Theta }{dt}=-\Theta \end{array}$$

Unsupervised STDP with homeostatic mechanisms results in meaningful, low-dimensional representations of information present in the hidden layers utilizing only local plasticity mechanisms in contrast to training the entire network with expensive gradient descent based learning algorithms. This allows the deep-LSM to extract temporal information over multiple time-scales with only local learning rules which is ideal for neuromorphic implementations (Neftci et al., 2017).

To summarize the information processing in the deep-LSM, the hidden layers capture dynamic information about the input signal over multiple times-scales. The WTA layers are trained to condense the high-dimensional hidden layer activity into a meaningful low-dimensional representation. This ensures that the inputs to each hidden layer provide useful information, while keeping the inputs to each hidden layer low-dimensional. This is important because the hidden layers rely on creating a high-dimensional representation of their input, by forming low-dimensional inputs it reduces the size of the deeper hidden layer which improves the scalability of the architecture.

### 3.3. Attention Mechanism

**Figure 3:**
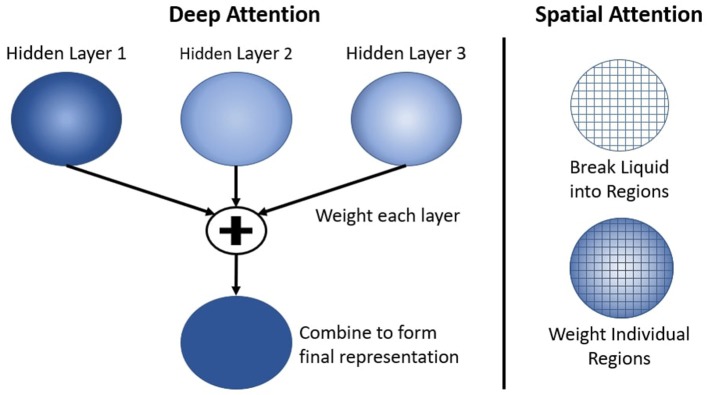
Attention applied to the cumulative deep-LSM network referred to as the deep attention network **(Left)** and spatially to a single hidden layer through the spatial attention network **(Right)**.

Another neural mechanism in the deep-LSM is the use of attention to selectively process information in the hidden layers as shown in Figure 3. As the size of the deep-LSM grows, attention allows the readout layer to perform classification with limited resources. Attention is applied by adding two separate single layer neural networks, which compute a weighted summation of all the hidden layers. This results in a single representation with the same dimensionality as one hidden layer being passed to the output layer. The attention networks receive the filtered state of the deep-LSM based on (4), *X*_*deep*−*LSM*_ = [*X*_1_, *X*_2_, …, *X*_*L*−1_, *X*_*L*_] where L is the number of hidden layers in the deep-LSM, to predict the appropriate attention coefficients.

First, the deep attention network predicts the importance of each layer in the deep-LSM. The attention network will predict a coefficient for each hidden layer in the deep-LSM based on the current state. The function of the deep attention network's operation is given by

(12) $$\begin{array}{l} A_{l}^{deep}=softmax_{l}\left(W^{A^{deep}}*X^{deep-LSM}\right) \end{array}$$

where *A*_*l*_ refers to the attention coefficient for the *l*^*th*^ hidden layer in the network such that $A^{deep}=\left[A_{1}^{deep},A_{2}^{deep},\ldots ,A_{L-1}^{deep},A_{L}^{deep}\right]$ and L represents the total number of layers and $W^{A_{l}^{deep}}$ are the learned weights of the deep attention network. A softmax function is used to assign a probability to each layer which represents the importance of that layer. Then, based on the attention coefficients, a weighted sum of all the hidden layers is computed to generate a final representation of the deep-LSM (*X*^*S*^) as shown in (13)

(13) $$\begin{array}{l} X^{S}=\overset{L}{\underset{l=1}{\sum }}A_{l}^{deep}*X_{l} \end{array}$$

Second, the spatial attention network will predict the importance of each neuron in the final representation *X*_*S*_. The second attention network receives the same input as the first attention network and will predict a coefficient $A_{n}^{Spatial}$ for every value in the final representation *X*^*S*^, this can be applied to every neuron or a population of neurons. This will assign a weight to each neuron/population, allowing the output layer to focus on a select subset of signals. The operation for computing $A_{n}^{Spatial}$ is given by

(14) $$\begin{array}{l} A_{n}^{Spatial}=\sigma \left(W_{n}^{A^{Spatial}}*X_{deep-LSM}\right) \end{array}$$

where each coefficient $A_{n}^{Spatial}$ is determined based on the learned weights for the *n*^*th*^ neuron in the spatial attention network, $W_{n}^{A^{spatial}}$, the state of the deep-LSM, and $A^{Spatial}=\left[A_{1}^{Spatial},A_{2}^{Spatial},\ldots ,A_{N-1}^{Spatial},A_{N}^{Spatial}\right]$ where N is the total number of neurons in a hidden layer. The coefficients in *A*^*Spatial*^ will then be used to produce a weighted representation of *X*^*S*^ where

(15) $$\begin{array}{l} X^{F}=X^{S}⊙A^{Spatial} \end{array}$$

where the final representation of deep-LSM's state *X*^*F*^, is computed by an element-wise multiplication between the spatial attention coefficients and their corresponding location in *X*^*S*^. *X*^*F*^ is then sent to the output layer which performs classification or prediction, given by (16)

(16) $$\begin{array}{l} y\left(n\right)=\sigma \left(W^{out}*X^{F}\right) \end{array}$$

where *y*(*n*) is the output of the readout layer based on the state *H*_*F*_ of the deep-LSM at time *t* = *n*.

## 4. Experiments

**Figure 4:**
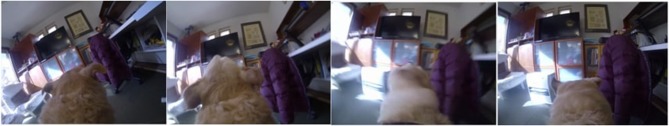
Sample video frame sequence from DogCentric dataset for the shake class [models tested with hand crafted features (HFC) and without HFCs are separated].

The proposed deep-LSM was benchmarked for video activity recognition using the DogCentric dataset (Iwashita et al., 2014). The DogCentric dataset consists of 209 videos recorded for ten different activities being performed by four different dogs from a first-person view point. A sample of the image frames for the shake class is shown in Figure 4. The videos possess rapid and erratic movement, similar to a person running around with a camera, making it challenging to process what is occurring. There is also an imbalance in the datasamples with unequal number of videos per class. To make a fair comparison with prior networks, samples for every class were distributed equally between training and test data, similar to Graham et al. (2017).

The video frame features were extracted with a pre-trained ResNet-50 architecture which were then reduced to 100 dimensions using principal component analysis. The 100-dimensional features for each frame were used as an input to the deep-LSM and LSM models for classification at the end of each video sequence. The framelength in the DogCentric dataset varies from 30 frames to 650, with an average of 157 frames per video. Results were averaged for 150 runs of each model. The deep-LSM outperformed state-of-the-art models shown in Table 1, including a single layer LSM with an equal number of neurons and an attention modulated readout layer. The parameters used to obtain the results presented are given in Table 2.

**Table 1:** Comparison of state-of-the-art accuracy results on the DogCentric dataset.

	**Approach**	**Accuracy**
HCF	GOFF + VIF + Log-C + Cuboids (Arabacı et al., 2018)	64.0%
	HOG+HOF+LBP+Cub.+Opt.Fl. (Iwashita et al., 2014)	60.5%
	ITF (Wang and Schmid, 2013; Piergiovanni et al., 2016)	67.7%
	ITF+CNN (Jain et al., 2014; Piergiovanni et al., 2016)	69.2%
	POT (Ryoo et al., 2015)	73.0%
	POT+ITF (Ryoo et al., 2015)	74.5%
	TDD (Wang et al., 2015; Piergiovanni et al., 2016)	76.6%
	TDD+Temp. Fil. (Piergiovanni et al., 2016)	79.6%
	TDD+Temp. Fil.+LSTM (Piergiovanni et al., 2016)	81.4%
No HCF	VGG+Max Pooling (Piergiovanni et al., 2016)	≈57.2%
	VGG+Mean Pooling (Piergiovanni et al., 2016)	59.9%
	VGG+Sum Pooling (Piergiovanni et al., 2016)	59.9%
	VGG+Temp. Fil.-Learned (Piergiovanni et al., 2016)	≈65.0%
	VGG+Temp. Fil.-Learned+LSTM (Piergiovanni et al., 2016)	≈65.0%
	CDN (VGG-16) (Graham et al., 2017)	75.8%
	CDN (ResNet-50) (Graham et al., 2017)	77.2%
	TCF (CaffeNet) (Kahani et al., 2017)	72.19%
	TCF (VGG-16) (Kahani et al., 2017)	77.79%
	TCS (VGG, TDD) (Kahani et al., 2017)	82.24%
	LFP (G+SD+GS) (Kwon et al., 2018)	82.5%
	**Deep-LSM (ResNet-50)**	**84.78%**
	LSM (ResNet-50)	76.5%

**Table 2:** Parameters used in proposed deep-LSM and standard LSM implementation.

**Parameter**	**Value**
Simulation timestep	1 ms
*V*_*th*_	16.5 mV
τ_*m*_	28 ms
*C*_*mem*_	1 pF
τ_*ref*_	4 ms
*D*^*H*^ (deep-LSM/LSM)	1,000/3,000
E:I Ratio	4:1
Synaptic Strength (EE/EI/II/IE)	3/3/1/4
λ (2) (EE/EI/II/IE)	3/3/3/3
C (2) (EE/EI/II/IE)	0.6/1/0.2/1
α (9) (E/I)	40/36
α (3)	0.007
β (3)	0.739
*D*^*W*^	50–100–150
*X*^*tar*^ (8)	25–50
τ_*trace*_ (4)	300 ms
α (9) (Synaptic scaling in WTA layer)	15
Θ (10)	1.5 mV
τ (11)	500 ms

**Figure 5:**
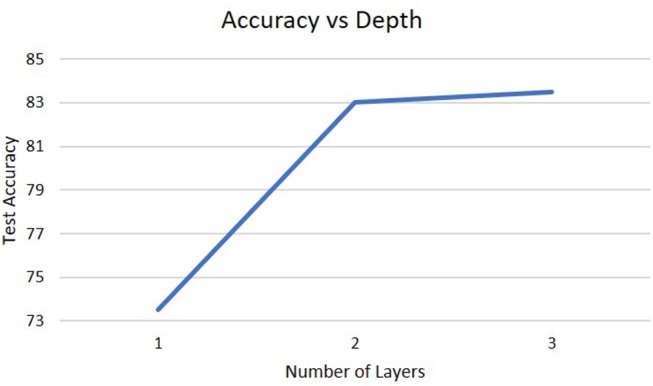
Accuracy on the DogCentric dataset as a function of the number of layers in the deep-LSM (each hidden layer has 1,000 neurons, while each encoding layer has 50 neurons).

To analyze the impact of different architectures in the deep-LSM, the network was studied for a different number of layers, for different sizes of the hidden layer, and for different sizes of the WTA layer. As shown in Figure 5, a single layer LSM is inferior to a deep-LSM with multiple layers and as the number of layers increases from three to five, the deep-LSM is better at processing the complex temporal information in the video.

**Figure 6:**
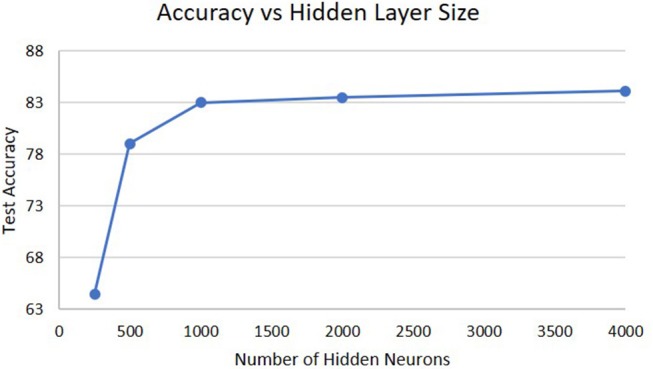
Accuracy on the DogCentric dataset as a function of hidden layer size in a 3-layer deep-LSM (each WTA layer has 50 neurons).

The next analysis was how the size of the hidden layer affects performance shown in Figure 6. Increasing the size of the hidden layers or the WTA layers does not result in much difference in performance. For the size of the hidden layer, it is already sufficient with 1,000 neurons to create a high-dimensional representation of the input for extracting temporal information and further increases do not result in any change. If we decrease the hidden layer size, eventually a point is crossed where the high-dimensional representation does not capture enough information about the input and the performance will drop. This can be seen from the degradation in accuracy as the hidden layer size decreases to 250 neurons.

**Figure 7:**
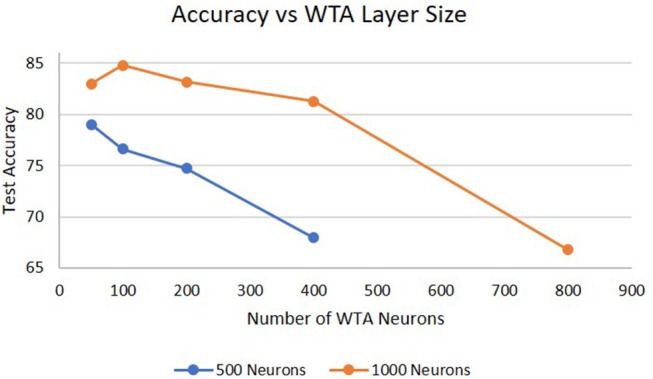
Accuracy on the DogCentric dataset as a function of WTA layer size in a 3-layer deep-LSM (each hidden layer has 1,000 neurons).

Lastly, Figure 7 shows the performance as a function of the size of the WTA layer. For a 1,000 neuron hidden layer, increasing the WTA layer size from 50 to 100 neurons shows an increase in performance because the WTA layer can capture more features describing the hidden layer. However, when the WTA layer size increases to 200 neurons the performance significantly drops. Similar results were observed for a 500 neuron hidden layer, which showed degradation in performance beyond 50 neurons. The reason for this is that there are now too many signals feeding into the next hidden layer which dominates the hidden layers dynamics, and because there is likely little information gained by the extra 100 neurons. We hypothesize that the optimal size of the WTA layer is dependent on the size of the hidden layer. With smaller hidden layers, there will be less features for the WTA layer to identify and learn so increasing the number of neurons does not have an impact on the information sent between layers. Another way to view this is as if one was doing principal component analysis on the hidden layers output, only the top few principal components would be needed to convey the important information between layers. In addition, the dimensionality of the WTA layer cannot be too close to the dimensionality of the hidden layer or it will negatively impact the information processing of deeper hidden layers. Another potential cause of this result is the hyper-parameters for the WTA layer are not optimal for allowing the network to efficiently learn at larger sizes (e.g., homeostatic mechanisms, training epochs).

### 4.1. Theoretical Efficiency for Neuromophic Implementations

To analyze the efficiency of the deep-LSM for on-device implementations, we study the deep-LSM in an application dependent framework for processing temporal information on embedded platforms. The first analysis is to compare the total number of synaptic connections as well as the types of training computations needed to assess the scalability and memory cost of the proposed model with respect to other recurrent neural networks. Table 3 reports of the number of synaptic connections based on the type of learning for three temporal networks with an equal number of neurons; the deep-LSM, a traditional LSM, and an standard LSTM. A hypothetical LSTM model is used as a baseline purely for scalability analysis on the basis of an equivalent number of neurons and does not consider architectures such as stacked LSTMs. The analysis is performed for a deep-LSM which consists of 100 input neurons, 3 hidden layers with 500 neurons, two winner-take-all layers with 50 neurons, two attention networks (one with 3 neurons for each hidden layer and one with 500 neurons for each location in the hidden layer), and a readout layer with 10 neurons, one for each class. To determine the synaptic connections in the LSM and LSTM networks, we consider them to possess recurrent layers with 1500 neurons (which is equivalent to the total number of neurons in the three deep-LSM hidden layers). In addition, we consider a similar attention-based readout layer for the LSM which would implement spatial attention with 1500 neurons. As can be seen in Table 3, the deep-LSM with attention requires 35.69% of the number of synapses as the LSM with attention, but 613% the number of synapses as a standard LSM. However, a deep-LSM without attention only has 77.86% as many synapses as a standard LSM. In comparison to the LSTM model, a deep-LSM with the proposed attention mechanism has 8.87% of the number of synaptic connections with a similar number of neurons.

**Table 3:** Number of synaptic connections trained with different learning rules and their memory consumption for the deep-LSM, LSM, and LSTM.

	**Backpropagation**	**Random**	**Unsupervised**	**Total**	**Memory (Gb)**
Deep-LSM	15,000	90,738	2,500	108,238	0.0035
Deep-LSM + Attention	759,500	90,738	2,500	852,738	0.0273
LSM	15,000	124,016	0	139,016	0.0044
LSM + Attention	2,265,000	124,016	0	2,389,016	0.0764
LSTM	9,615,000	0	0	9,615,000	0.3077

From the table we can see that between the deep-LSM and LSM, with a similar readout layer (attention or single-layer), the deep-LSM shows a reduction in the number of synaptic weights. These calculations account for the sparsity values which had been used in our simulations, which was 95% sparsity in the input connections of both models, 89.24% sparsity in the deep-LSM hidden layers, and 95% sparsity in the LSM. Though the degree of sparsity varied in the hidden layer between the deep-LSM and LSM, they were generated from the same network hyper-parameters in (2). The difference arises from the deep-LSM having a smaller reservoir size which reduced the number of long-range connections which tended to not form a connection. In comparison to the LSTM, the deep-LSM with attention only has 7.93% as many trainable synaptic connections. In addition the deep-LSM attention weights are trained by a gradient descent algorithm which does not require sequential back-propagation-through-time. As for the connections trained through STDP, they only require an accumulation of a neurons activity (which is done per neuron rather than per synapse) and is only invoked when a neuron fires rather than every synapse being updated on each training operation. Therefore, the deep-LSM's training is computationally much lighter than the LSTM with respect to both the number and type of operations, and total number of trainable synapses.

The number of operations during inference and training in each model is reported in Table 4, which we computed for the deep-LSM and LSM based on our implementation, and for the LSTM based on derivation of the training and inference phase in Chen (2016) and are summarized in Table 5 for inference and Table 6 for training. These estimates calculate the number of multiplications needed in the specified models assuming that the number of additions would be similar and ignoring the cost of neuron functions and hyper-parameters. Based on the results, the deep-LSM with attention only has 8.45% of the number of computations as a vanilla LSTM and only 0.65% the number of computations without the attention module. In comparison between a deep-LSM and LSM, when an attention-based readout layer is used the deep-LSM has 64.84% fewer operations and significantly lower number of weight updates. Without attention the deep-LSM shows a 16.2% decrease but a slightly higher number of weight updates due to the unsupervised connections. Thus, separating the attention layer from the analysis, the deep-LSM shows a slight reduction in computational cost compared to the standard LSM.

**Table 4:** Number of synaptic operations for a single frame of training data (for STDP synapses, only one post-synaptic neuron can win at any time frame).

**Network**	**# Multiplications (FP)**	**# Multiplications (BP)**	**# Weight updates**
Deep-LSM	110,700	15,000	17,500
Deep-LSM + Attention	857,200	773,506	760,500
LSM	135,000	15,000	15,000
LSM + Attention	2,386,500	2,251,500	2,251,500
LSTM	9,619,500	9,675,000	9,615,000

**Table 5:** Computation of the number of multiplications needed during inference (FP).

**Network**	**# Multiplications (Forward pass)**
Deep-LSM	*S*_*in*_ * *N* * *H*_*d*_ + 2(*l* − 1) * *S*_*in*_ * (*W* * *H*_*d*_) + *l* * *S*_*R*_ * (*H*_*d*_ * *H*_*d*_) + *l* * *H*_*d*_ * *O*
Deep-LSM + A	*S*_*in*_ * *N* * *H*_*d*_ + 2(*l* − 1) * *S*_*in*_ * (*W* * *H*_*d*_) + *l* * *S*_*R*_ * (*H*_*d*_ * *H*_*d*_) + *l*(*H*_*d*_ * *A*) + *l* * *H*_*d*_ + *H*_*d*_ + *H*_*d*_ * *O*
LSM	*S*_*in*_ * *N* * *H* + *S*_*R*_ * *H* * *H* + *H* * *O*
LSM + A	*S*_*in*_ * *N* * *H* + *S*_*R*_ * *H* * *H* + *H* * *H* + *H* * *O*
LSTM	4 * (*N* * *H* + *H* * *H*) + 3 * *H* + *H* * *O*

*N is the dimensionlaity of the input, H_d_ is the dimensionality of the deep-LSM hidden layers, W is the dimensionality of the WTA layers, A is the combined dimensionality of both attention networks, O is the dimensionality of the output, l is the number of layers, and H is the dimensionality of the hidden layer in the LSM and LSTM. For the LSM and deep_L_SM, S_in_ is the input sparsity and S_R_ is the hidden layer sparsity. Note “+ A” refers to inclusion of the attention-based readout layer*.

**Table 6:** Computation of the number of multiplications needed during training (Backward Pass).

**Network**	**# Multiplications (Backward pass)**
Deep-LSM	*l* * (*O* * *H*_*d*_)
Deep-LSM = A	3 * (*O* * *H*_*d*_) + *A* * *H*_*d*_ * *l* + 2 * *H*_*d*_ + 2 * *l* + *l* * 2 * *H*_*d*_
LSM	*H* * *O*
LSM + A	*H* * *O* + *H* * *H*
LSTM	2 * (*H* * *O*) + 30 * *H* + 4 * (*H* * *N*) + 4 * (*H* * *H*)

Another important feature for algorithms on embedded platforms is robustness to device noise. To assess the robustness of the deep-LSM, we mimic device noise in a neuromemristive system by adding Gaussian noise on every read and write operation as in Soures et al. (2018). As shown in Table 7, the networks performance suffers very little degradation due to the presence of noise.

**Table 7:** Performance on the DogCentrric dataset for a 3 layer deep-LSM when Gaussian noise is introduced.

**Model (3 layers)**	**Accuracy**	**Standard deviation**
Deep-LSM	82.9	6.78
Deep-LSM (with noise)	81.92	10.08

Finally, the energy consumption (estimated based on Han et al., 2016, for 45 nm technology node) of the proposed deep-LSM is compared with that of an LSM and LSTM. The energy is estimated by calculating the number of addition (0.9pJ) and multiplication (3.7pJ) operations (of 32-bit precision) for training and inference, and the number of synaptic weights stored in DRAM (360pJ).

Based on Table 8, it can be observed that the deep-LSM is more energy efficient than an LSTM during training, inference, and consumes less memory. When compared to the LSM, we see that the deep-LSM is more energy efficient when using an equivalent readout layer.

**Table 8:** Energy portfolio of deep-LSM, LSM, and LSTM for inference, training, and memory.

	**Inference**	**Training**	**Weights**	**Total**
	**Energy (μJ)**	**Energy (μJ)**	**Energy (μJ)**	**Energy (μJ)**
Deep-LSM	0.5092	0.069	38.9657	39.5439
Deep-LSM + Attention	3.9431	3.5581	306.9857	314.4869
LSM	0.621	0.069	50.0458	50.7358
LSM + Attention	10.9779	10.3569	860.0458	881.3806
LSTM	44.24	55.56	5866.6	5966.4

*Estimates are for a 45 nm CMOS technology node (Han et al., 2016)*.

From this analysis, we conclude that the deep-LSM is a computationally lite model for processing temporal information with a fraction of the memory and compute operations compared to other popular recurrent neural network architectures. The deep-LSM has several features which result in its higher performance with respect to other algorithms. The first key feature of the deep-LSM is its modular reservoirs which create the deep architecture for the network. By using a modular approach, the deep-LSM reduces the size of the recurrent matrices needed by the network and also demonstrates a much better capability at extracting information over multiple time-scales as shown by the large increase in performance over traditional RC approaches. The second key feature of the deep-LSM is the use of spiking WTA layers in between hidden layers. This allows to extract meaningful features to propagate through the network and helps alleviate the dependence of traditional RC approaches on their initialization. The WTA layers learn their features through an unsupervised local learning rule which allows the network to learn and optimize its connections at a lower cost than gradient descent. Additionally, because STDP is a local learning rule the layers can be trained without waiting for information to be propagated backwards speeding up the training time and allowing the WTA layers to be updated in parallel. Finally, the last feature of the deep-LSM which contributes to its performance are the attention layers. Due to the large savings in total number of synaptic connections and reduced amount of training due to random connections, the deep-LSM can implement the attention layers while still maintaining an overall reduction in the number of synapses.

## 5. Conclusions

We proposed a new approach for performing spatio-temporal tasks on a budget. The proposed deep-LSM has promising results in video activity recognition achieving 84.78% on a representative dataset and surpasses state-of-the-art algorithms in accuracy. More importantly, the deep-LSM consumes significantly lower synaptic memory storage and computational resources. Edge devices naturally benefit from this computationally light algorithm and the following benefits ensue.

1. Edge intelligence framework: Suitable for real-time on-device learning and inference.
2. Local unsupervised plasticity mechanisms: Enable fine-grained tuning to trade-off compute complexity vs. accuracy.
3. Broaden applicability of RC approaches to complex temporal problems that require integration of information over multiple time-scales.
4. An overall reduction in energy consumption and memory requirements compared to current recurrent networks.

## Data Availability

Publicly available datasets were analyzed in this study. This data can be found here: http://robotics.ait.kyushu-u.ac.jp/~yumi/db/first_dog.html.

## Author Contributions

NS as the first author performed the experiments and was responsible for writing and creating figures and tables. DK was responsible for writing and guidance in the design and experiments.

### Conflict of Interest Statement

The authors declare that the research was conducted in the absence of any commercial or financial relationships that could be construed as a potential conflict of interest.
